# Kontinuierliches intraoperatives Neuromonitoring (cIONM) in der Kopf-Hals-Chirurgie – eine Übersicht

**DOI:** 10.1007/s00106-020-00823-2

**Published:** 2020-03-10

**Authors:** P Stankovic, J. Wittlinger, R. Georgiew, N. Dominas, S. Hoch, T. Wilhelm

**Affiliations:** 1 Klinik für HNO-Heilkunde, Kopf‑/Hals- und plastische Gesichtschirurgie, Sana Kliniken Leipziger Land, Rudolf-Virchow-Straße 2, 04552 Borna, Deutschland; 2 Universitätsklinik und Poliklinik für Hals-Nasen-Ohren-Heilkunde, Kopf- und Hals-Chirurgie, Martin-Luther-Universität Halle-Wittenberg, Halle/Saale, Deutschland; 3 Universitätsklinik für Hals‑, Nasen und Ohrenheilkunde, Phoniatrie und Pädaudiologie, Philipps-Universität Marburg, Marburg, Deutschland; 4 Medizinische Fakultät, Philipps-Universität Marburg, Marburg, Deutschland

**Keywords:** Kleinhirnbrückenwinkel, Thyreoidektomie, Intraoperatives neurophysiologisches Neuromonitoring, N. facialis, N. vagus, Cerebellopontine angle, Thyroidectomy, Intraoperative neurophysiological monitoring, Facial nerve, Vagal nerve

## Abstract

Obwohl die Geschichte des intraoperativen Neuromonitorings (IONM) bereits in das 19. Jahrhundert zurückdatiert werden kann, hat sich diese Methode bis vor Kurzem nicht von der reinen Differenzierung des Nervs weiterentwickelt. Erst das kontinuierliche IONM (cIONM) ermöglichte die durchgehende Analyse der Reizamplituden und -latenzen, welche mittlerweile ebenfalls in die Software gängiger Monitoringsysteme integriert wurde. Zielsetzung des cIONM ist ein Real-Time-Monitoring des Nervenstatus während des Eingriffs, um so drohende Nervenverletzung erkennen und verhindern zu können und die postoperative Funktion des Nervs vorhersehbar zu erhalten. Trotz einiger Nachteile wie falsch-positiver oder -negativer Alarme, technischer Artefakte und seltener Nebenwirkungen bleibt das cIONM ein gutes Hilfsmittel, das noch weiterentwickelt wird. In der Literatur sind sowohl aktive (acIONM) als auch passive (pcIONM) Reiz- und Ableitmethoden des cIONM beschrieben. Derzeit gängige Anwendungsgebiete des cIONM umfassen die Schilddrüsenchirurgie mit der kontinuierlichen Stimulation des N. vagus sowie die Chirurgie des Kleinhirnbrückenwinkels (KHBW) mit dem Monitoring des N. facialis; hierbei werden neben kontinuierlicher Stimulation auch die Entladungsmuster des Nervs analysiert. Des Weiteren ist in die Chirurgie des KHBW das kontinuierliche Monitoring des Hörnervs etabliert.

## Abstract (en)

Although the history of intraoperative neuromonitoring (IONM) dates back to the 19th century, the method did not evolve further than the mere differentiation of nerves until recently. Only the development of continuous IONM (cIONM) has allowed for non-stop analysis of excitation amplitude and latency during surgical procedures, which is nowadays integrated into the software of almost all commercially available neuromonitoring devices. The objective of cIONM is real-time monitoring of nerve status in order to recognize and prevent impending nerve injury and predict postoperative nerve function. Despite some drawbacks such as false-positive/negative alarms, technical artefacts, and rare adverse effects, cIONM remains a good instrument which is still under development. Active (acIONM) and passive (pcIONM) methods of cIONM are described in literature. The main fields of cIONM implementation are currently thyroid surgery (in which the vagal nerve is continuously stimulated) and surgery to the cerebellopontine angle (in which the facial nerve is either continuously stimulated or the discharge signal of the nerve is analyzed via pcIONM). In the latter surgery, continuous monitoring of the cochlear nerve is also established.

Intraoperatives Neuromonitoring (IONM) zielt darauf ab, gefährdete neuronale Strukturen anatomisch und funktionell zu erhalten und so postoperativ temporäre und dauerhafte Paresen zu vermeiden. Modernes IONM ermöglicht nicht nur Nervenidentifizierung, sondern auch die Erkennung potenziell schädlicher Manipulationen; so kann anhand der intraoperativen Informationen eine Vorhersage der postoperativen Funktion erfolgen. Im Kopf-Hals-Bereich ist das IONM in der HNO-Heilkunde, Neurochirurgie und Schilddrüsenchirurgie etabliert.

## Hintergrund

Die Geschichte des intraoperativen Neuromonitorings (IONM) reicht bis ins Jahr 1898 zurück, als Dr. Fedor Krause aus Berlin die monopolare faradische Stimulation bei der Neurektomie des N. vestibulocochlearis als Therapie eines dekompensierten Tinnitus zur Identifikation des N. facialis einsetzte [13]: Hierbei stimulierte er den Gesichtsnerv und stellte visuell fest, dass Kontraktionen der Gesichtsregion erfolgten, insbesondere des M. orbicularis oculi sowie der die Nase und den Mund versorgenden Äste. Die Durchbruchsjahre für das IONM waren die 1960er-Jahre, als Flisberg und Lindholm [7] das IONM in die Schilddrüsenchirurgie einführten sowie Parsons und Hilger in verschiedenen Arbeiten Stimulatoren des Gesichtsnervs für die Parotis- und Ohrchirurgie entwickelten [10, 20].

In den letzten Jahrzehnten wurde die Nutzung des IONM in vielen Kliniken zum Standard, wobei es hier hauptsächlich zur Identifikation des Nervs bei der Präparation in der Nähe desselben eingesetzt wurde. Die hauptsächlichen Anwendungsgebiete waren die Schilddrüsenchirurgie, bei der der N. vagus und der N. recurrens überwacht wurden, sowie Parotischirurgie und Eingriffe im Kleinhirnbrückenwinkel (KHBW), bei denen der Gesichtsnerv überwacht wurde.

Beim intermittierenden IONM wird die meiste Zeit „nicht gehört, was der Nerv zu sagen hat“

Grundprinzip ist hierbei, dass während der Operation die intakte Nervenfunktion durch distale Reizung und Registrierung der myogenen Antwort überprüft und nachgewiesen wird. Darüber hinaus kann so bei fraglichen neuralen Strukturen eine Identifikation des Nervs erfolgen. Die Sonde wird auf den Nerv gelegt, wodurch bei jeder Reizung des Nervs visuelle oder akustische Reize erzeugt werden. Diese Art der Anwendung kann als intermittierendes IONM (iIONM) definiert werden. Beim intermittierenden IONM wird die meiste Zeit „nicht gehört, was der Nerv zu sagen hat“.

In der HNO-Heilkunde wird iIONM hauptsächlich in der Parotischirurgie eingesetzt. Einige Autoren berichteten über niedrigere Raten postoperativer Fazialisparesen bei Anwendung von iIONM [16, 27, 35], andererseits gibt es ebenfalls Studien, die das Gegenteil behaupten [4, 9, 40]. In den Vereinigten Staaten gibt es keine klare Empfehlung, ob ein Kopf-Hals-Chirurg iIONM verwenden sollte, wenn er Operationen an der Parotis vornimmt. Dies führt dazu, dass derzeit 40 % der HNO-Ärzte in den USA routinemäßig kein iIONM verwenden, sondern sich auf ihre chirurgischen Fähigkeiten und Kenntnisse anatomischer Orientierungspunkte verlassen [17]. Eine kürzlich veröffentliche Metaanalyse, bei der Parotidektomien mit und ohne iIONM verglichen wurden, zeigte, dass die Häufigkeit einer unmittelbar postoperativen Fazialisparese nach Parotidektomie bei Verwendung von iIONM signifikant geringer war als bei intraoperativer Nichtüberwachung [34].

Ziel des cIONM ist die Echtzeitüberwachung des Nervenstatus während der chirurgischen Manipulation

In den letzten Jahren wurde das IONM durch die Einführung eines sog. kontinuierlichen intraoperativen Neuromonitorings (cIONM) weiterentwickelt. Dies zielte darauf ab, in Echtzeit den Nervenzustand während des gesamten Verlaufs der Operation zu erfassen und – noch wichtiger – so die postoperative Funktion vorherzusagen. Dies wurde entweder aktiv (acIONM) oder passiv (pcIONM) durchgeführt (Tab. 1). Beim acIONM wird der Nerv kontinuierlich während des gesamten Eingriffs stimuliert. Hierzu wird eine Reizelektrode direkt auf dem Nerv oder in der Nähe desselben platziert. Dieses Prinzip wird ebenfalls bei der transkraniellen Nervenstimulation oder im Fall der Überwachung des N. VIII mithilfe akustischer Stimuli eingesetzt. Das aktive cIONM wurde in der Schilddrüsenchirurgie [5, 8, 12, 14, 21, 25, 30, 32, 33], bei Eingriffen in der hinteren Schädelgrube [1, 2, 37, 41] und bei Operationen von Gefäßanomalien des Gesichts [38] entwickelt, um N. vagus, facialis und vestibulocochlearis zu schonen.

**Table 1:** 

**Aktives kontinuierliches intraoperatives Neuromonitoring (acIONM)**
*N. facialis (N. VII)*
– Direkt (KHBW-Chirurgie)
– Perkutan (Chirurgie der vaskulären Malformationen)
– Transkraniell (elektrische Stimulation des Tractus corticobulbaris bei der KHBW-Chirurgie)
*N. vestibulocochlearis (N. VIII)*
– Direkt akustisch (KHBW-Chirurgie)
*N. vagus (N. X)*
– Direkt (Schilddrüsenchirurgie)
**Passives kontinuierliches intraoperatives Neuromonitoring (pcIONM)**
*N. facialis (N. VII)*
– Freilaufendes, prozessiertes Entladungs-EMG

*EMG *Elektromyogramm, *KHBW *Kleinhirnbrückenwinkel

An dieser Stelle ist es wichtig, iIONM von cIONM zu unterscheiden, da einige Autoren iIONM manchmal als kontinuierlich bezeichnen, was zu Verwirrung führen kann. Während des iIONM ist der Patient zwar ständig an das Monitoringsystem angeschlossen, die Informationen, die der Chirurg erhält, sind jedoch nur lückenhaft, da sie erst bei aktiver Reizung neuraler Strukturen als Antwortpotenziale registriert werden: In diesem Setting ist die Identifikation des Nervs im umgebenden Gewebe nur dann möglich, wenn der Chirurg die Stimulationssonde aktiv verwendet. Es wird keine Analyse der Nervenamplituden und -latenzen durchgeführt, was dazu führt, dass während des größten Teils der operativen Maßnahmen „das, was der Nerv zu sagen hat, nicht wahrgenommen wird“. Während des cIONM hingegen wird eine ununterbrochene Analyse der Amplitude und der Latenz der neuralen Aktivität in das Monitoringsystem „eingespeist“, wodurch eine computergestützte Analyse ermöglicht wird.

## Nutzen des cIONM

### acIONM des N. vagus in der Schilddrüsenchirurgie

In der Literatur sind 5 verschiedene Modalitäten des acIONM beschrieben (Tab. 1). Ein aktives cIONM zur Funktionsüberwachung des N. recurrens wird durch die Positionierung einer Elektrode am N. vagus zwischen der A. carotis communis und der V. jugularis interna ermöglicht.

**Figure 1:**
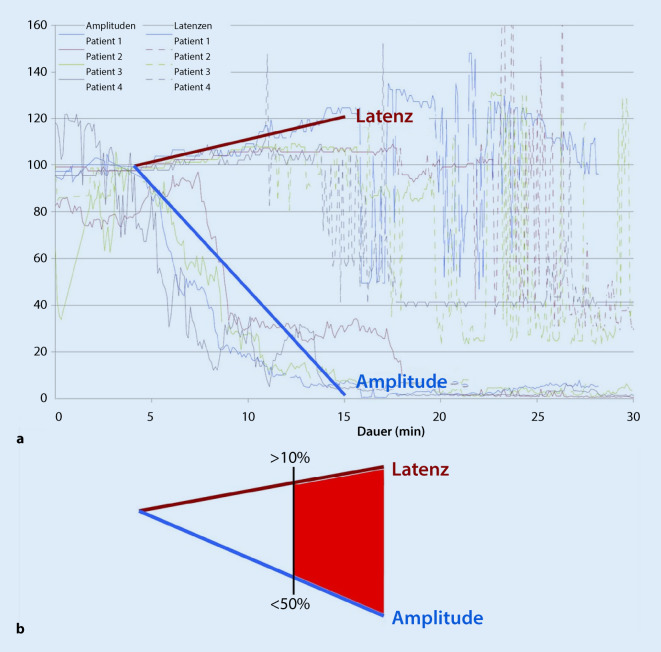

Hier wurde ein Muster einer drohenden Nervenschädigung identifiziert [32]: Ein kombinierter Abfall der Amplitude um mehr als 50 % und eine verlängerte Latenz um mehr als 10 %, was als multiples kombiniertes Ereignis („multiple combined event“, mCE) bezeichnet wurde, geht einem vollständigen Signalverlust voraus (LOS, „loss of signal“, Amplitude <100 μV), was einen postoperativen Stimmbandstillstand voraussagt (Abb. 1).

Ein Amplitudenabfall >50 % korrelierte mit einer postoperativen Nervenbeeinträchtigung

Wenn sich das mCE nicht zu einem LOS entwickelte, blieb die postoperative Funktion des Nervs normal. Daher kann die postoperative Parese vermieden werden, wenn dem Operateur während des Eingriffs ein mCE durch das Monitoringsystem angezeigt wird und er die zum Ereignis führenden Manipulationen (z. B. Traktion) korrigiert. Dadurch können drohende Nervenschädigungen vermieden werden.

Auf der Grundlage, dass das neurophysiologische Muster der Nervenschädigung während der cIONM „real-time“ verfolgt werden kann, wurde eine Studie publiziert, in der cIONM und iIONM bei 1526 aufeinanderfolgenden Schilddrüsenoperationen verglichen wurden, [33]. Das cIONM zeigte hier eine statistisch signifikante Überlegenheit in Bezug auf die permanente Stimmbandlähmung. Bei Patienten der cIONM-Gruppe wurden 77 mCE festgestellt, von denen 63 (82 %) vom Operateur durch Veränderung des chirurgischen Vorgehens aktiv rückgängig gemacht werden konnten [33].

Des Weiteren zeigten Schneider et al. in einer multizentrischen Studie, dass eine Signalerholung mit einer Amplitude ≥50 % der Baseline-Amplitude nach einem LOS mit einer intakten postoperativen Stimmlippenfunktion korreliert [31]. Diese Information erleichtert die Entscheidung, ob bei bilateral vorgesehener Schilddrüsenresektion die Resektion der Gegenseite vorgenommen werden kann, sollte ein LOS auf der zuerst operierten Seite registriert worden sein. Wenn es zu einer Signalerholung ≥50 % der Baseline-Amplitude kommt, könne den Autoren zufolge ohne weiteres die Operation der kontralateralen Seite in gleicher Sitzung erfolgen.

### Direktes acIONM des N. facialis in der Chirurgie des KHBW

Eine direkte Stimulation des Gesichtsnervs während einer Operation an der hinteren Schädelgrube wurde von Amano 2011 beschrieben [1], bei der er den Nerv direkt an der Wurzelaustrittszone mit einer durch Wattepads fixierten Kugelelektrode stimulierte. Hierbei wurden signifikante Unterschiede in Bezug auf die letzte maximale Amplitude sowie das Amplitudenerhaltungsverhältnis (letzte Amplitude am Ende der Resektion im Vergleich zur Basisamplitude) zwischen den Gruppen unterschiedlicher Fazialisparesen festgestellt. Darüber hinaus wurde gezeigt, dass Patienten mit guten postoperativen funktionellen Ergebnissen anhand der House-Brackmann(HB)-Skala sowie Patienten mit einer langfristigen postoperativen Verbesserung des HB-Grads statistisch höhere Amplitudenerhaltungsraten aufwiesen [1]. Daher bewerteten die Autoren das acIONM bei Operationen in der hinteren Schädelgrube im Hinblick auf die Erhaltung der Gesichtsnervenfunktion als positiv.

### acIONM bei der Chirurgie der vaskulären Malformationen des Gesichts

Die extrakranielle Methode des Neuromonitorings des Gesichtsnervs stellt die perkutane Stimulation dar. Ulkatan et al. verwendeten 2 monopolare Elektromyographie(EMG)-Nadeln, die perkutan in der Nähe das Foramen stylomastoideum positioniert wurden, bei der Operation von Gefäßmissbildungen des Gesichts [38]. Bei 161 vorwiegend jungen Patienten (Durchschnittsalter 14 Jahre) ermöglichte das acIONM eine präoperative Nervenkartierung, hauptsächlich bei Patienten, bei denen das Gesichtsödem aufgrund einer präoperativen Sklerosierung die Muskelzuckungen maskierte; die Nadelplatzierung war bei allen Operationen ohne Komplikationen möglich [38]. Am Beginn der Operation wurde ein Basiswert der Amplitude festgelegt, Amplitudenabsenkungen von <50 % im Vergleich zum Basiswert waren hier der Hinweis an den Operateur, die Manipulationen zu stoppen, bis sich die Amplitude normalisiert. Intraoperative Nervenläsionen wurden in allen 3 Fällen korrekt erkannt, und es wurde eine End-to-End-Neuroraphie durchgeführt; Im Follow-up erholten sich alle Patienten über einen längeren Zeitraum bis HB Grad I/II.

### acIONM als transkranielle Stimulation bei Eingriffen im KHBW

Die transkranielle Mehrfachimpuls-Elektrostimulation (TES) des kortikobulbären Trakts ist die Methode zur kontinuierlichen Überwachung der Funktion des Gesichtsnervs durch Analyse von muskelmotorisch evozierten Potenzialen („facial nerve motor-evoked potentials“, FNMEP) bei Eingriffen in der hinteren Schädelgrube. Die Stimulationssonde in Form einer Becherelektrode wird über dem Schädel platziert. Dieses Verfahren verwendet Cluster von 3–4 Stromimpulsen, die eine supramaximale Stimulation (100–400 V) mit einem Interpulsintervall von 1–2 ms und einer Clusterfrequenz von 5,6–3,3 × 10^−3^ Hz aufweisen [6].

Amplitudenerholung von ≥50 % nach LOS korreliert mit einer normalen postoperativen Nervenfunktion

Dong et al. zeigten, dass kein Patient mit einer Endamplitude von 50 % oder mehr im Vergleich zur Baseline-Amplitude eine deutliche Verschlechterung der Gesichtsnervenfunktion postoperativ aufwies [6].

### acIONM des N. vestibulocochlearis bei Eingriffen im KHBW

Das Neuromonitoring des N. vestibulocochlearis unter Verwendung von auditorisch evozierten Hirnstammpotenzialen (BAEP) während einer Operation im KHBW kann ebenfalls als acIONM bezeichnet werden. Während der Präparation in der Nähe des N. VIII werden dem Ohr über Ohrstöpsel kontinuierlich akustische Klicks von 100–110 dB angeboten. Die in der BAEP abgeleiteten Wellen JEWETT I und V liefern aufgrund ihrer Konstanz die nützlichsten Informationen, teilweise kann auch die Welle III zur Auswertung herangezogen werden. Das andere Ohr wird durch weißes Rauschen von 60–70 dB vertäubt. Ein ähnliches Verfahren, welches bei den gleichen Operationen verwendet wird, ist die Elektrocochleographie (ECochG): das acIONM ermöglicht hier die Ableitung einer Nervenantwort, die der JEWETT-Welle I des BAEP entspricht, jedoch mit einer signifikant höheren Amplitude. Hierbei ist auch das „compound action potential“ (CAP) zu beachten. Diese Methode nutzt eine Elektrode, die entweder zwischen dem Tumor und dem Wurzeleintritt des Nervs in den Hirnstamm [41] oder distal zum Tumor [11] platziert wird. BAEP, ECochG und CAP sind komplementäre Methoden, die sich gegenseitig nicht ausschließen, sondern gemeinsam gleichzeitig angewendet werden können.

Das BAEP-basierte acIONM hat zuverlässige Ergebnisse bei der Vorhersage der postoperativen Hörfunktion gezeigt. Beispielsweise teilten Neu et al. [19] Patienten in 4 Gruppen ein, die mit acIONM unter Verwendung von BAEP überwacht wurden. Alle Patienten mit stabiler Welle V (Muster 1) zeigten eine eindeutige Erhaltung des Hörvermögens, während alle Patienten mit irreversiblem abruptem Verlust des BAEP (Muster 2) trotz direkt postoperativer Erhaltung des Hörvermögens in 2 Fällen ihr Hörvermögen im Verlauf verloren. Alle Patienten mit einem irreversiblen progressiven Verlust von Welle I oder Welle V (Muster 3) erlitten ebenfalls trotz direkt postoperativer Erhaltung des Hörvermögens in 2 Fällen schließlich einen definitiven Hörverlust. Die Fälle mit intraoperativ reversiblem BAEP-Verlust (Muster 4) zeigten ein variables kurz- und langfristiges Hörergebnis [19].

Yamakami et al. wendeten bei ihren Eingriffen BAEP und CAP gleichzeitig an [41]: Zuverlässige BAEP-Werte bezogen auf Welle V konnten nur bei 41 % der Patienten abgeleitet werden, während bei 91 % der Patienten ein reproduzierbares CAP ohne Artefakte registriert wurde. Alle Patienten, deren CAP nach Abschluss der mikrochirurgischen Tumorresektion erhalten war, zeigten postoperativ ein brauchbares Gehör. Somit wurde eine 100%ige Spezifität und Sensitivität nachgewiesen [41].

### pcIONM des Gesichtsnervs bei Eingriffen im KHBW

Neben dem aktiven kontinuierlichen IONM haben sich Methoden zur kontinuierlichen Überwachung entwickelt, die ausschließlich auf der Analyse der Entladungsmuster beruhen, die während der Operation auftreten: Dies kann als passives cIONM bezeichnet werden. Ein solches „freilaufendes EMG“ wird in der Neurochirurgie zur Überwachung des N. facialis eingesetzt. Prass und Lüders beschrieben „spikes“, „bursts“ und 3 Arten von „trains“ nach Analyse des EMG-Signals von 30 Patienten nach KHBW-Chirurgie [22]. Eine anhaltende periodische EMG-Aktivität wurde als „train“ bezeichnet. Das Vorhandensein von „A-Trains“ [22], ein hochfrequentes und niedrigamplituden-sinusförmiges EMG-Muster, wurde in einer anderen Studie mit einem schlechteren postoperativen HB-Score korreliert [26]. Es ist erst Prell und seiner Gruppe gelungen, eine Software zu entwickeln, die in Echtzeit im Operationssaal eingesetzt wurde [23, 24], was die Echtzeitquantifizierung der „train time“ ermöglichte. So konnte den Operateur über die „kumulative Schädigung“ des Nervs informiert werden. Dies ermöglichte, in Echtzeit über die bevorstehende Nervenverletzung zu informieren, wodurch der Operateur die wahrscheinliche postoperative Nervenfunktion abschätzen und die operative Strategie aktiv ändern konnte, um eine weitere Verschlechterung der Nervenfunktion zu vermeiden.

Modernes IONM ermöglicht intraoperativ eine Abschätzung der postoperativen Nervenfunktion

Hieraus wurde der Nervenzustand in Analogie mit einer Verkehrsampel bewertet: Der Nervenzustand ist „grün“, sobald die kumulative Zeit aller „trains“ („train time“) unter 0,125 s beträgt, woraus folgert, dass die Präparation sicher fortgeführt werden kann. Mit Anstieg der „train time“ bis 2,5 s wechselt das Ampellicht auf „gelb“, was eine erhöhte Vorsicht gebietet, da eine solche Dauer der „train time“ in 1/4 der Fälle mit Fazialisparesen vom Grad HB III korreliert war. Ein weiterer Anstieg der „train time“ über 2,5 s führte dazu, dass das Ampellicht auf „rot“ wechselte, da hier eine deutlich verschlechterte Fazialisfunktion zu erwarten ist. In diesem Fall müsste die Manipulation am Nerv abgebrochen und der operative Plan wieder hinterfragt werden. Als Konsequenz des „roten“ Lichts wurden z. B. der Zugangswinkel und/oder der Ort der Präparation geändert, Nimodipin intraoperativ verabreicht sowie in einigen Fällen die Operation abgebrochen und eine Revision geplant.

## Sicherheit

Beim cIONM steht neben der Spezifität und Sensitivität die Sicherheit hinsichtlich möglicher Nervenläsionen durch die repetitiven Reizungen oder sonstiger Kollateralwirkungen im Vordergrund. Das passive cIONM ist in dieser Hinsicht unbedenklich, da es auf der reinen Analyse der gemessenen Muskelaktivität beruht und keine aktiven Stimulationen stattfinden. Dies ist beim aktiven cIONM jedoch nicht der Fall, da hier aktive Stimulationen angewendet werden. Daher müssen hier Sicherheitsaspekte geprüft und beachtet werden.

Das aktive cIONM wurde in großen Studien zur Schilddrüsenchirurgie überwiegend als sicheres Verfahren beschrieben [8, 32, 33]. In einer prospektiven Studie wurde die Sicherheit des acIONM nachgewiesen, da keine Herzfrequenzvariabilität und immunmodulatorischen Effekte durch kontinuierliche Stimulation des N. vagus festgestellt wurden [8]. Dies wurde auch in einer früheren Studie derselben Autorengruppe beobachtet, in der ein deutlicher Einfluss von acIONM auf das Gleichgewicht des autonomen Nervensystems bestand, ohne dass sich die Herzfrequenz, der Rhythmus oder die hämodynamischen Parameter veränderten [39]. In einer Studie an 102 Patienten stellten Phelan et al. fest, dass es weder zu unerwünschten Amplituden- oder Latenzveränderungen noch zu unerwünschten gastrointestinalen, kardialen oder pulmonalen Nebenwirkungen kam [21]. Ein aktives cIONM wurde auch bei Patienten mit fortgeschnittenem atrioventrikulärem Block ohne Nebenwirkungen eingesetzt [28].

In einer experimentellen Studie von Lee et al. an 13 Schweinen wurde acIONM unter Verwendung einer automatisierten Periodenstimulation angewendet, um die Kraft zu untersuchen, die für eine Traktionsverletzung des N. recurrens erforderlich ist [15]. Die Nerven wurden hierbei gedehnt, bis ein Signalverlust auftrat. Eine Erholung des EMG-Signals zeigten alle Nerven 7 Tage nach dem Experiment, was darauf hindeutete, dass acIONM allein keine strukturelle Schädigung des Nervs hervorruft [15], der sich in der Folge auch wieder erholen kann.

Es wurde jedoch ebenfalls über einige Nebenwirkungen berichtet [3, 18, 36]. Bei einem Patienten wurde eine reversible vagale Neuropraxie durch die APS®-Stimulationselektrode („automatic periodic stimulation“, Fa. Medtronic ENT, Jacksonville/FL, USA) mit sichtbarer perineuraler Ekchymose gesehen: Der Nerv konnte nach dem Ereignis nicht mehr stimuliert werden. In diesem Fall kam es zu einer postoperativen kurzfristigen Parese, die sich aber nach einem Monat völlig erholte. In derselben Veröffentlichung wurde eine schwerwiegende hämodynamische Instabilität (Bradykardie und Hypotonie) nach dem Einsetzen des acIONM bei einem jungen gesunden Patienten ohne Vorgeschichte von Herzereignissen festgestellt. Der Effekt konnte durch Entfernen der Elektrode prompt rückgängig gemacht werden. Beim Wiedereinsetzen der Elektrode wurde nochmals der gleiche Effekt beobachtet, der durch nochmaliges Entfernen der Elektrode wieder reversibel war. Die Patientenrekrutierung in der Studie wurde nach diesen beiden unerwünschten Ereignissen abgebrochen [36].

In 2 Studien, in denen zusammen fast 250 Nerven analysiert wurden, wurde die identische Wahrscheinlichkeit von 2 % für eine Verletzung des Vagusnervs aufgrund der Platzierung der APS®-Elektrode angegeben [3, 18]. Die Ereignisse führten jedoch nicht zu einer permanenten postoperativen Rekurrensparese.

### Sicherheit des acIONM bei Kindern

Die Sicherheit des acIONM wurde auch in einigen Studien bei Kindern untersucht. So verwendeten z. B. Bozinov et al. eine transkranielle Elektrostimulation, um bei 21 Patienten (5 Monate bis 15 Jahre alt, Durchschnittsalter 5,5 Jahre) kontinuierlich die motorisch evozierten Potenziale des Gesichtsnervs (FNMEP) zu überwachen. FNMEP waren auch in dieser jungen Patientengruppe anwendbar und mit ähnlichen Ergebnissen sicher, die bei der Vorhersage der postoperativen Gesichtsnervenfunktion bei Erwachsenen in anderen Untersuchungen erzielt wurden. Der HB-Grad blieb prä- und postoperativ in 23 von 24 Operationen gleich. Das Vorhandensein von FNMEP beeinflusste die chirurgische Strategie insofern, als in den Fällen, in denen die direkte Nervenstimulation keine Muskelreaktion hervorrief, die Tumorresektion fortgeführt wurde [2]. Ein weiteres Beispiel für das acIONM bei Kindern ist die bereits erwähnte Studie von Ulkatan et al., in der die perkutane Stimulation in 201 Operationen bei 161 Patienten im durchschnittlichen Alter von 14 Jahren angewendet wurde [38]. Die Sicherheit des acIONM bei Schilddrüsenoperationen wurde ebenfalls bei einer großen Studie mit 105 Kindern belegt [29].

## Einschränkungen

Abgesehen von den erwähnten seltenen Nebenwirkungen aufgrund der Elektrodenplatzierung sind einige andere Einschränkungen zu nennen. Nach Erfahrung der Autoren muss das Problem der häufig auftretenden technischen Artefakte und falsch-positiven oder -negativen Alarme stets beachtet werden. Dies kommt v. a. beim cIONM mit Echtzeitnervenüberwachung zum Tragen. Allen genannten Studien ist gemeinsam, dass man sich eines niedrigen positiven Vorhersagewerts bewusst sein muss. Daher kann beispielsweise in der Schilddrüsenchirurgie ein falsch-positiver Alarm einen unnötig verzögerten Eingriff an der kontralateralen Seite bedingen.

Bisher wurden keine Studien mit cIONM bei der Parotischirurgie durchgeführt

Abgesehen von seltenen retrospektiven Studien, in denen cIONM mit iIONM in der Schilddrüsenchirurgie verglichen wurde, gibt es keine Veröffentlichungen, in denen diese neuartige Methode mit der konventionellen iIONM in den anderen zuvor genannten Anwendungsgebieten verglichen wird. Prospektive randomisierte Studien sind hier dringend erforderlich, um den behaupteten Nutzen nachzuweisen. Bisher wurden keine Studien mit cIONM bei der Parotischirurgie durchgeführt.

Einige der genannten Methoden bleiben „Einzelstudienberichte“, d. h. sie haben sich in der klinischen Praxis nicht etabliert (s. Abschnitt „Direktes acIONM des N. facialis in der Chirurgie des KHBW“ und Abschnitt „acIONM bei der Chirurgie der vaskulären Malformationen des Gesichts“). Bei anderen Studien wurden nur die zuletzt erfasste Amplitude und die Basisamplitude zur statistischen Auswertung herangezogen (s. Abschnitt „Direktes acIONM des N. facialis in der Chirurgie des KHBW“) oder die Frequenz der kontinuierlichen Stimulation betrug lediglich eine Stimulation in 3–5 min (s. Abschnitt „acIONM als transkranielle Stimulation bei Eingriffen im KHBW“), was die Notwendigkeit und auch den Nutzen einer kontinuierlichen Stimulation infrage stellt.

## Fazit für die Praxis

- Kontinuierliches intraoperatives Neuromonitoring (cIONM) als eine neuartige Methode hat die Erkennung einer drohenden Nervenverletzung und die damit verbundene Änderung der operativen Strategie ermöglicht.
- Darüber hinaus wurde die Vorhersage der postoperativen Nervenfunktion auf Basis von cIONM im Vergleich zu intermittierendem intraoperativem Neuromonitoring (iIONM) verfeinert.
- cIONM eröffnet dem Bereich Neuromonitoring eine neue Dimension.
- Dabei handelt es sich um ein Verfahren, das Chirurgen bei der Durchführung von chirurgischen Manövern in unmittelbarer Nähe neuronaler Strukturen helfen kann.
- Es kann eine ausgefeilte Operationstechnik nicht ersetzen, bietet jedoch zuverlässige und sichere Unterstützung.
- In zahlreichen Studien und Tierversuchen wurde die Sicherheit des aktiven cIONM nachgewiesen.
- Die bisherigen Ergebnisse unterstützen die Anwendung des cIONM auch in der Parotischirurgie, in der sich bisher weder das aktive noch das passive cIONM etablieren konnten.
